# Testing Continuous Age as an Effect Modifier of the Association Between Diminished Ikigai and Psychological Distress in Two Long-Term Disaster Contexts in Japan

**DOI:** 10.3390/diseases14100364

**Published:** 2026-10-02

**Authors:** Tetsushi Omori, Michiko Iwai

**Affiliations:** 1 Faculty of Language Studies, Teikyo University, 359 Otsuka, Hachioji-shi, Tokyo 192-0395, Japan; 2 School of Marine Technology, Tokyo University of Marine Science and Technology, 2-1-6 Etchujima, Koto-ku, Tokyo 135-8533, Japan

**Keywords:** ageing, continuous age, ikigai, psychological distress, GHQ-28, disaster recovery, effect modification

## Abstract

Background/Objectives: Mental-health research often represents chronological age as a threshold or adjustment variable, although psychosocial associations may vary across adulthood. We tested whether continuous age modified the association between a context-specific single-item indicator of diminished ikigai and psychological distress in two Japanese communities with long-term disaster consequences. The ikigai indicators differed by context and were not treated as equivalent multidimensional measures. Methods: Secondary cross-sectional analyses used Miyakejima 2020/21 (*N* = 158; complete-case *n* = 147) and Okuma 2024 (*N* = 134; 111 valid 28-item General Health Questionnaire (GHQ-28) classifications; complete-case *n* = 110). Age-by-ikigai interactions were primary; fixed <65/≥65 analyses and robustness checks were secondary or exploratory. Results: In Miyakejima, the estimated interaction odds ratio (OR) was 2.00 per 10 years (95% confidence interval [CI] [1.03, 3.91], *p* = 0.042; likelihood-ratio *p* = 0.029), with substantial uncertainty at age extremes; the threshold interaction was unsupported. In Okuma, low/absent ikigai was associated with GHQ-28 high-risk status (OR = 12.94, 95% CI [4.96, 33.8], *p* < 0.001), while age modification was not detected (interaction OR = 0.88, 95% CI [0.44, 1.76], *p* = 0.711). The historical Miyakejima reanalysis also did not provide detectable evidence of corresponding modification. Conclusions: Within these context-specific samples, continuous-age modelling yielded preliminary evidence of heterogeneity in Miyakejima not captured by a 65-year threshold. Larger longitudinal studies should evaluate this signal’s precision and transferability.

## 1. Introduction

Purpose, meaningful activity, and social participation are associated with mental well-being in later adulthood [1,2,3,4,5]. Age is often handled in this literature by restricting samples to older adults, dividing participants at a conventional cut-point, or entering age only as an adjustment variable. These approaches answer useful descriptive or adjustment questions, but they do not directly test whether the strength of a psychosocial association changes across the adult age range.

Ikigai is a culturally situated Japanese concept commonly translated as a sense that life is worth living or as purpose in life [6,7,8]. Multidimensional scales and longitudinal studies link ikigai with psychological, social, and physical well-being [9,10], and recent studies have examined ikigai in middle-aged and older adults and in national adult samples [11,12,13]. The present source surveys did not administer a multidimensional ikigai scale. They used single questions about felt ikigai, so the focal exposure is interpreted here as a context-specific indicator of perceived ikigai rather than as a complete measurement of the broader construct.

Long-term disaster research documents persistent psychological distress and heterogeneous recovery trajectories [14,15,16,17]. Studies of older adults exposed to human-induced disasters also underscore the importance of considering disaster context in interpreting vulnerability [18,19], while Japanese disaster cohorts report that social isolation, home loss, community conditions, and other post-disaster circumstances are associated with mental health or recovery years after disaster [20,21,22,23,24,25]. Earlier work in the present research settings documented psychological distress among Fukushima evacuees [26] and examined mental-health factors in Miyakejima [27]. Whether the ikigai–distress association itself varies with continuous age long after evacuation and return, however, has not been directly tested in these datasets.

The primary analytic question for this secondary reanalysis was therefore whether continuous age modified the association between diminished ikigai and 28-item General Health Questionnaire (GHQ-28) high-risk status within each contemporary context. We used a two-sided age × ikigai interaction test and did not assume a direction of modification. The <65/≥65 specification was retained only as a secondary comparison with conventional ageing practice. Firth models, influence diagnostics, nonlinearity checks, one-at-a-time perturbation analyses, alternative Okuma ikigai coding, and the archived 2013/14 Miyakejima wave were exploratory sensitivity analyses. The datasets were analysed separately; cross-context contrasts are descriptive, and no context × ikigai × age interaction was tested. The intended contribution is methodological: to examine what continuous-age modelling can reveal that a fixed older-adult threshold may obscure in context-specific, long-term disaster datasets.

## 2. Materials and Methods

### 2.1. Design and Context

This study comprised secondary cross-sectional analyses of existing community surveys and an exploratory historical reanalysis of an earlier Miyakejima wave. The source datasets included GHQ-28 psychological distress, age, ikigai, and related factors. The present reanalysis focused on continuous age as an effect modifier. Miyakejima 2020/21 was conducted approximately 20 years after the 2000 volcanic eruption and island-wide evacuation. Okuma 2024 was conducted 13 years after the Great East Japan Earthquake and Fukushima Daiichi nuclear accident among adults who had returned to live in the town. The 2013/14 Miyakejima archive was used only as an exploratory historical sensitivity analysis; the Miyakejima waves were unlinked repeated cross-sectional samples.

### 2.2. Participants and Sampling

The Miyakejima source survey targeted adults aged ≥20 years listed in the Basic Resident Register for the Tsubota District. Postal fieldwork was conducted from 28 October to 30 November 2020. Because postal return was limited, in-person distribution and collection were added on 16–18 March and 3–6 April 2021. Of 406 questionnaires distributed, 201 were returned (49.5%); 43 respondents without direct eruption experience were excluded, leaving *N* = 158. GHQ classification and verified age were available for all 158 respondents. The de-identified analytic file did not retain participant-level collection-mode or collection-period identifiers, so age distributions could not be compared between the postal and in-person phases.

The Okuma dataset included 134 adult returnees surveyed from February to September 2024. With town cooperation, questionnaires were distributed door to door to adults aged ≥20 years who had lived in Okuma on 11 March 2011 and were residing in the town at the time of survey. Of 250 distributed questionnaires, 134 valid responses were obtained (53.6%); 111 respondents had valid GHQ-28 classifications. Municipal statistics recorded 269 residents in the administrative returnee category on 31 May and 282 on 30 September 2024 [28,29]. These counts contextualise the contemporaneous returnee population but are not a study denominator because administrative and study eligibility definitions differ. Former residents who remained displaced or relocated were outside the sampling frame.

The archived 2013/14 Miyakejima survey included 204 adults and was used only for exploratory historical analyses. Sample sizes for all analyses were fixed by the available source datasets; no prospective sample-size calculation was possible for this secondary reanalysis. Sample characteristics, missingness, and primary analytic denominators are summarised in Table 1.

### 2.3. Measures

Psychological distress was assessed with the authorised Japanese GHQ-28 [30,31]. Items were scored 0-0-1-1, yielding totals from 0 to 28; scores ≥ 6 were classified as GHQ high risk in accordance with established Japanese guidance [32]. Analyses used complete GHQ-28 responses without imputation or prorating. Internal consistency of the GHQ-28 was high in both contemporary samples (Cronbach’s α = 0.89 in Miyakejima and 0.902 in Okuma).

In Miyakejima, perceived ikigai was assessed with one question asking whether respondents felt a sense of ikigai in their current daily life. Responses were often, sometimes, seldom, and not at all; often/sometimes were classified as present and seldom/not at all as absent. Valid responses were available for 147 participants (95 present, 52 absent). In Okuma, one question used six ordered categories; the three affirmative categories were classified as present and the three negative categories as low or absent. Valid responses were available for 133 participants. These items were treated as related, context-specific indicators of perceived ikigai rather than as equivalent measures of a multidimensional construct.

Age was recorded in years. For the primary models, age was centred at 65 years and scaled per 10-year increase. Secondary variables (<65 versus ≥ 65 years) were derived for comparison with conventional Japanese ageing statistics [33], not because age 65 was assumed to be a biological or psychological threshold.

### 2.4. Analysis

Analyses were implemented in R version 4.5.1 (R Foundation for Statistical Computing, Vienna, Austria); Firth models used the logistf package version 1.26.1. The primary complete-case datasets comprised 147 Miyakejima respondents and 110 Okuma respondents with valid GHQ-28 classification, context-specific ikigai status, and continuous age. Within each context, we fitted an additive logistic model and a model containing diminished ikigai × continuous age. The interaction odds ratio (OR) represents the multiplicative change in the context-specific ikigai OR for each 10-year increase in age. These models estimate observed associations rather than causal effects; no covariate-adjusted model is interpreted as a causal effect estimate. Interaction models were compared with their additive counterparts using likelihood-ratio tests (LRTs). Confidence intervals (CIs) were emphasised because the available information for interaction estimation was limited, particularly where age observations were sparse.

The <65/≥65 analyses were secondary. GHQ high-risk rates were cross-tabulated by ikigai and age group and are summarised descriptively in Table 2; threshold interaction models are reported in Supplementary Table S10. Because several subgroup cells were small, these percentages and tests are descriptive and should not be used to infer an abrupt age threshold.

Exploratory sensitivity analyses assessed estimate stability rather than providing formal confounding control. Firth bias-reduced models examined finite-sample estimation and separation-related instability. One-at-a-time models added source-survey variables selected as empirically relevant contextual factors potentially related to the observed association, including health, socioeconomic conditions, social relationships, participation, and disaster-related concerns [3,4,5,20,21,22,23,24,25,34,35,36]. These perturbation analyses do not establish control of confounding and cannot overcome selection bias or measurement error. Quadratic-age extensions and influence diagnostics were also exploratory; failure to detect a quadratic term was not treated as proof of linearity. *p* values for secondary and sensitivity analyses were not adjusted for multiplicity. Given the number of exploratory checks and the primary Miyakejima *p* value near 0.05, chance cannot be excluded.

Okuma additionally used the original six-category ikigai response as an ordered predictor and a boundary-exclusion sensitivity omitting the intermediate affirmative category. The archived 2013/14 Miyakejima data were analysed with Firth regression because separation occurred in the older, ikigai-absent cell. Model-based probabilities and representative-age estimates were retained as descriptive illustrations, with observed age density and uncertainty shown explicitly. Full sensitivity results are provided in the Supplementary Material.

### 2.5. Generative Artificial Intelligence Use

ChatGPT (OpenAI; GPT-5.6 Sol) was used for language refinement, document formatting, and assistance with R code syntax. It was also used to cross-check arithmetic and transcription of model output after analyses had been run by the authors. It did not select models, make autonomous analytic decisions, or create, alter, impute, or fabricate study data. All reported analyses and outputs were executed or independently verified by the authors against the source data and statistical output.

### 2.6. Ethical Considerations

The Miyakejima 2020/21 source survey was approved by the Research Ethics Committee of Teikyo University, Hachioji Campus on 19 August 2020; the committee assigned no approval number. The Okuma 2024 source survey was approved by the same committee (Approval No. 2023-8; 25 October 2023). The 2013/14 Miyakejima source survey was approved by the External Funding Management and Administration Committee of the Faculty of Business Administration, Yokohama National University; that committee assigned no approval number. Participation in the source surveys was voluntary, and written informed consent was obtained. The present manuscript reports secondary analyses of de-identified existing data and involved no new recruitment, data collection, or intervention.

## 3. Results

### 3.1. Miyakejima 2020/21

In the overall Miyakejima sample (*N* = 158), 81 respondents were GHQ high risk (51.3%). The primary continuous-age model used *n* = 147 complete cases. At age 65 years, the estimated ikigai-absence OR was 1.23 (95% CI [0.58, 2.57], *p* = 0.590), and the age OR among ikigai-present respondents was 0.87 per 10 years (95% CI [0.66, 1.16], *p* = 0.347). The estimated ikigai absence × age interaction was OR = 2.00 per 10 years (95% CI [1.03, 3.91], *p* = 0.042); adding the interaction improved fit over the additive model (LRT *p* = 0.029; Table 3). The confidence interval was wide and close to the null at its lower bound, so this result is treated as preliminary evidence of an age-dependent association rather than as a definitive age gradient.

**Figure 1:**
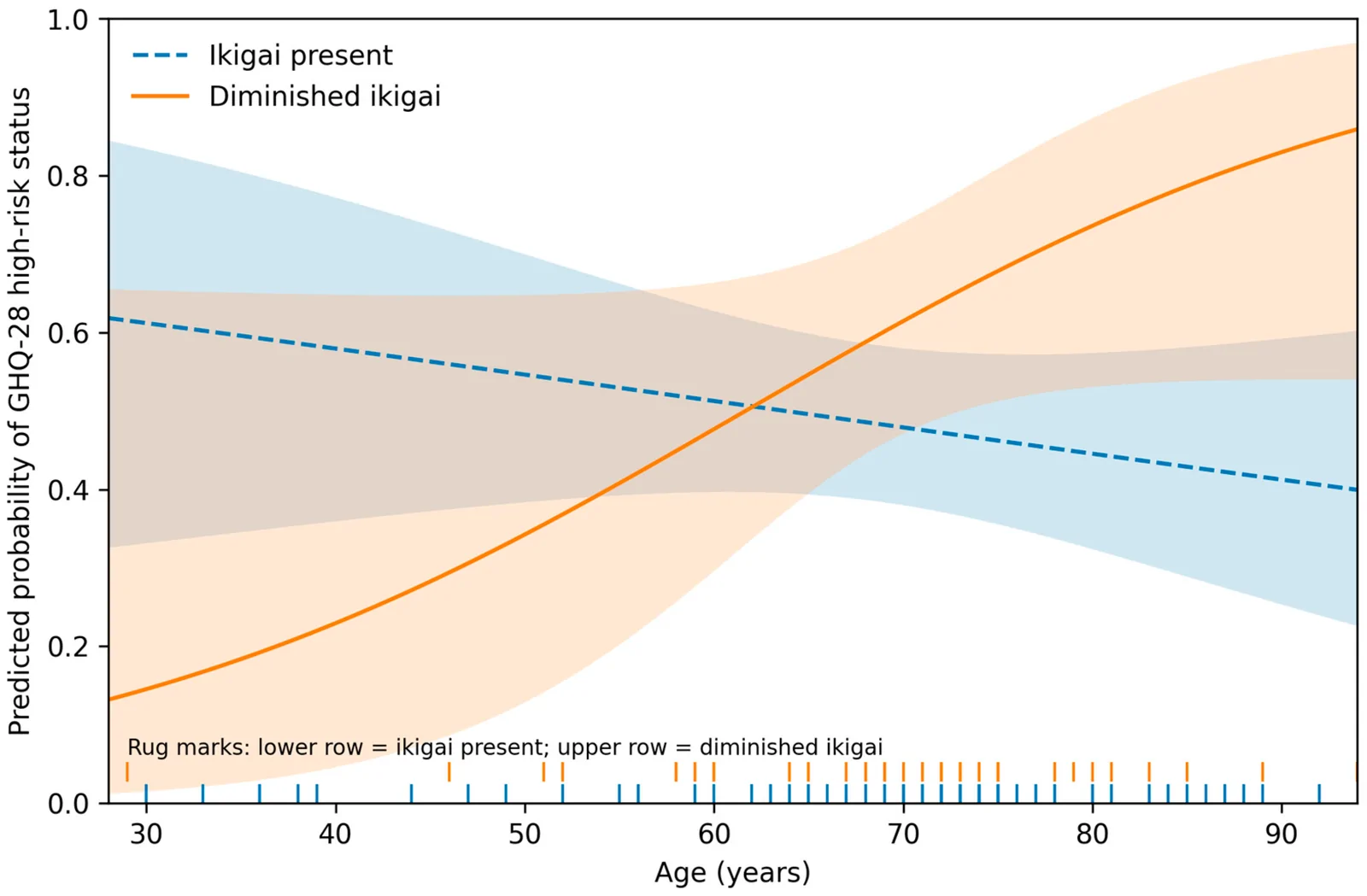
Predicted probability of 28-item General Health Questionnaire (GHQ-28) high-risk status by age and ikigai status in Miyakejima, 2020/21. Predictions are from the primary logistic interaction model (*n* = 147). Shaded bands indicate 95% confidence intervals; rug marks show observed ages in the primary complete-case sample using the corresponding legend colours (lower row = ikigai present; upper row = diminished ikigai). Estimates near the age-range extremes are based on sparse data and are correspondingly imprecise.

Model-based conditional ORs were 0.61 at age 55, 1.23 at 65, 2.45 at 75, and 4.91 at 85 years, but precision decreased toward the age extremes (Supplementary Table S8). Figure 1 therefore displays 95% confidence intervals together with rug marks for observed ages in the primary complete-case sample. Group-specific age distributions are reported in Supplementary Table S11.

Exploratory stability checks gave interaction estimates in the same direction. Firth estimation yielded an interaction OR of 1.88 per 10 years (95% profile penalised-likelihood CI [1.04, 3.80], *p* = 0.037), and the sex-adjusted Firth estimate was 1.90 (95% profile penalised-likelihood CI [1.05, 3.83], *p* = 0.035). One-at-a-time perturbation models produced interaction ORs from 1.92 to 2.59, and leave-one-out estimates remained above 1. These checks assess numerical stability; they do not resolve limited power, possible selection bias, measurement error, residual confounding, or multiplicity (Supplementary Tables S1, S4 and S7).

### 3.2. Okuma 2024

In Okuma, 62 of 111 respondents with valid GHQ-28 data were high risk (55.9%). The primary complete-case models used *n* = 110. In the additive model, low/absent ikigai was strongly associated with GHQ high-risk status (OR = 12.94, 95% CI [4.96, 33.8], *p* < 0.001), although the wide confidence interval indicates substantial uncertainty about the magnitude. Because the outcome was common, this OR must not be interpreted as a prevalence ratio or risk ratio.

**Figure 2:**
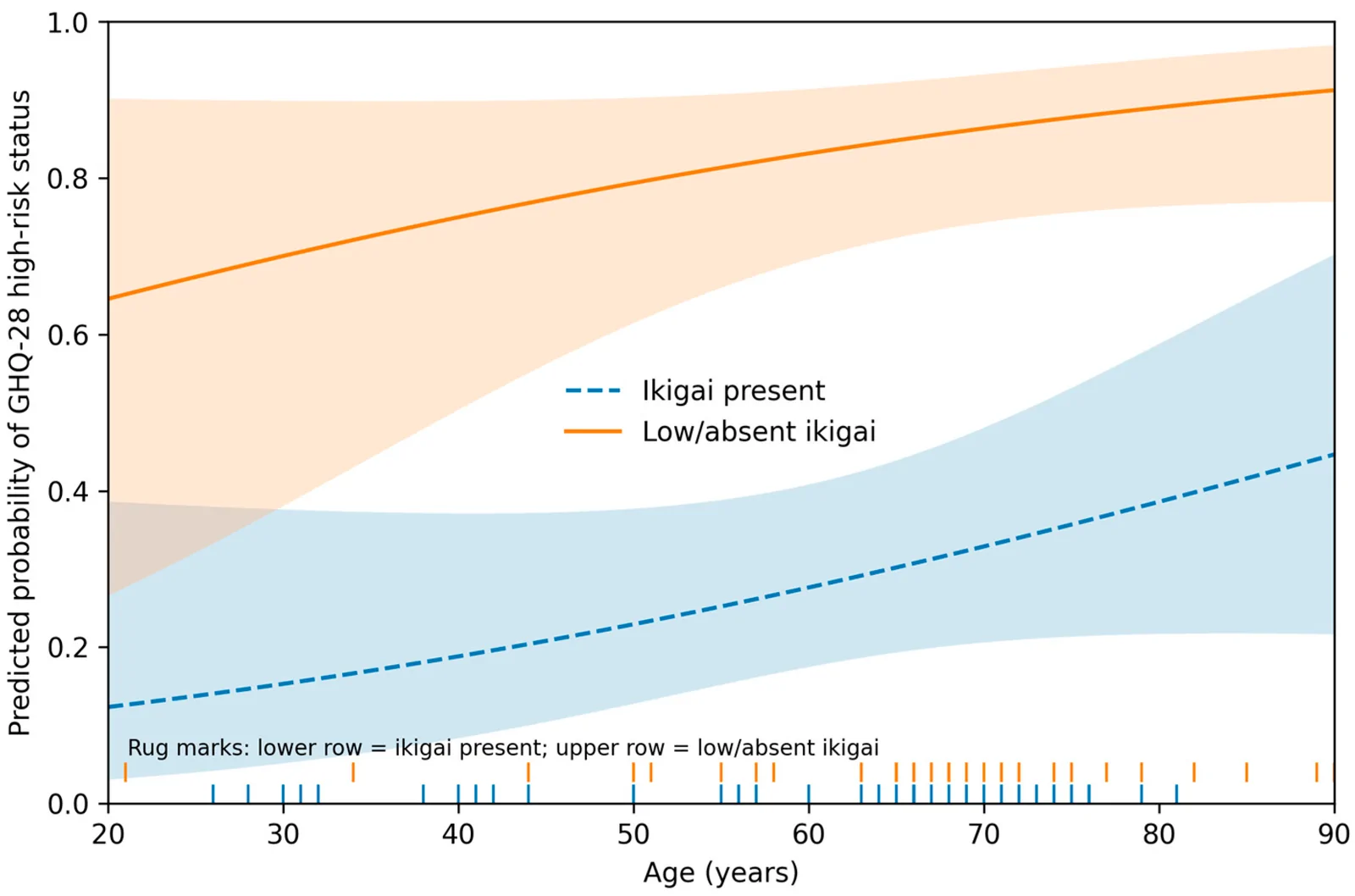
Predicted probability of 28-item General Health Questionnaire (GHQ-28) high-risk status by age and ikigai status in Okuma, 2024. Because the age × ikigai interaction was unsupported, predictions are shown from the additive logistic model (*n* = 110). Shaded bands indicate 95% confidence intervals; rug marks show observed ages in the primary complete-case sample using the corresponding legend colours (lower row = ikigai present; upper row = low/absent ikigai). The figure describes fitted associations and does not imply age-related effect modification.

In the interaction model, the low/absent-ikigai OR at age 65 years was 12.72 (95% CI [4.87, 33.25], *p* < 0.001), the age OR among ikigai-present respondents was 1.34 per 10 years (95% CI [0.89, 2.03], *p* = 0.163), and the age × ikigai interaction was OR = 0.88 (95% CI [0.44, 1.76], *p* = 0.711; LRT *p* = 0.708; Table 3). These data therefore did not provide detectable evidence of age-related effect modification; they do not establish that modification is absent. To avoid visually implying an unsupported interaction, Figure 2 presents probabilities from the additive model, with 95% confidence intervals and observed-age rug marks.

Exploratory Firth and leave-one-out interaction estimates also did not provide detectable evidence of modification. Alternative ikigai coding showed that the magnitude of the main association depended on how the single item was represented: retaining all six ordered categories yielded an additive OR of 3.28 per one-category shift toward less felt ikigai (95% CI [2.01, 5.33], *p* < 0.001), whereas the boundary-exclusion binary contrast yielded OR = 20.57 (95% CI [6.20, 68.27], *p* < 0.001). The age interactions remained unsupported under both codings (Supplementary Table S9). These coding sensitivities reinforce caution in interpreting the magnitude of the binary Okuma OR.

### 3.3. Secondary Threshold and Historical Analyses

Table 2 reports descriptive GHQ high-risk rates by conventional age group and context-specific ikigai status. Several cells are small, so the rates are presented without emphasizing subgroup significance tests. Formal threshold interaction models are reported in Supplementary Table S10 and did not provide detectable evidence of effect modification in either contemporary context (Miyakejima LRT *p* = 0.350; Okuma LRT *p* = 0.802). Thus, the preliminary Miyakejima continuous-age signal should not be interpreted as an abrupt change at age 65.

**Table 2:** Descriptive 28-item General Health Questionnaire (GHQ-28) High-Risk Rates by Context-Specific Ikigai Status and Age Group.

Context	Group	High Risk/Valid *N*	High-Risk Rate
Miyakejima	All ikigai-present	46/95	48.4%
Miyakejima	All ikigai-absent	31/52	59.6%
Miyakejima	<65, ikigai-present	13/25	52.0%
Miyakejima	<65, ikigai-absent	7/14	50.0%
Miyakejima	≥65, ikigai-present	33/70	47.1%
Miyakejima	≥65, ikigai-absent	24/38	63.2%
Okuma	All ikigai-present	16/57	28.1%
Okuma	All low/absent ikigai	45/53	84.9%
Okuma	<65, ikigai-present	6/25	24.0%
Okuma	<65, low/absent ikigai	11/14	78.6%
Okuma	≥65, ikigai-present	10/32	31.3%
Okuma	≥65, low/absent ikigai	34/39	87.2%

Note. Rates are descriptive. Some age-by-ikigai cells are small; corresponding percentages are therefore imprecise. Formal threshold interaction models are reported in Supplementary Table S10. GHQ-28 = 28-item General Health Questionnaire.

**Table 3:** Primary Continuous-Age Logistic Interaction Models Predicting 28-item General Health Questionnaire (GHQ-28) High-Risk Status.

Context	*N*	Predictor	OR	95% CI	Wald *p*	Interaction LRT *p*
Miyakejima	147	Ikigai absence at age 65 years	1.23	[0.58, 2.57]	0.590	
Miyakejima	147	Age per 10 years among ikigai-present	0.87	[0.66, 1.16]	0.347	
Miyakejima	147	Ikigai absence × age per 10 years	2.00	[1.03, 3.91]	0.042	0.029
Okuma	110	Low/absent ikigai at age 65 years	12.72	[4.87, 33.25]	<0.001	
Okuma	110	Age per 10 years among ikigai-present	1.34	[0.89, 2.03]	0.163	
Okuma	110	Low/absent ikigai × age per 10 years	0.88	[0.44, 1.76]	0.711	0.708

Note. Age was centred at 65 years and scaled per 10 years. LRT *p* values compare the interaction model with the corresponding additive model. Threshold analyses are separated into Supplementary Table S10. OR = odds ratio; CI = confidence interval; LRT = likelihood-ratio test; GHQ-28 = 28-item General Health Questionnaire.

The archived 2013/14 Miyakejima reanalysis showed strong ikigai–distress associations but did not provide detectable evidence of age modification. The exploratory continuous-age Firth interaction was OR = 0.85 per 10 years (95% profile penalised-likelihood CI [0.36, 2.23], *p* = 0.703), with a similar sex-adjusted estimate. Because the 2013/14 and 2020/21 samples were unlinked and the contemporary Okuma population differs substantially from Miyakejima, these patterns are descriptive across contexts and periods rather than evidence of temporal change or formal between-context differences.

## 4. Discussion

### 4.1. Main Findings and Uncertainty

The central result is methodological and context-specific. In Miyakejima 2020/21, continuous-age modelling produced a positive age × ikigai interaction that was not captured by the conventional 65-year threshold. The estimate was imprecise (OR = 2.00, 95% CI [1.03, 3.91]) and arose from a primary complete-case sample of 147; it should therefore be treated as preliminary evidence of an estimated age-dependent association. Okuma showed a strong association between low/absent ikigai and distress, but its *n* = 110 interaction analysis did not provide detectable evidence of age modification. The historical Miyakejima sensitivity analysis also did not detect modification. The present data therefore do not support a general lifespan mechanism.

The robustness analyses help assess whether the Miyakejima estimate is driven by a single modelling choice or influential observation, but they do not convert a context-bounded cross-sectional estimate into definitive evidence. Firth estimation addresses separation and some finite-sample estimation problems; it does not remedy limited precision or power, selection into the sample, residual confounding, or measurement error. Likewise, the exploratory perturbation and influence analyses are not independent replications. Their role is to describe stability around the primary estimate.

### 4.2. Methodological Contribution of Continuous-Age Modelling

The study does not establish that ikigai becomes more important with age in general. It shows that a continuous interaction test can ask a different question from an older-adult cut-point. A threshold model can fail to detect graded heterogeneity, while an age-adjusted model cannot test effect modification at all. In Miyakejima, the continuous and threshold specifications led to different inferential signals. In Okuma, neither specification detected modification. These contrasting within-context results support using continuous age as an analytic option when the scientific question concerns graded effect modification, while retaining explicit uncertainty about functional form and power.

The available data do not support flexible estimation of the age function. The absence of a significant quadratic extension does not prove linearity on the log-odds scale. Future studies with larger samples and denser age coverage should prespecify interaction hypotheses, use harmonised measures, and consider flexible age functions or splines when the information available supports them.

### 4.3. Contextual Interpretation of the Ikigai Indicator

Ikigai is culturally situated and broader than the single items used here [6,7,8,9,10]. The present analyses therefore concern reported felt ikigai, not a validated multidimensional ikigai score. Within long-term disaster contexts, diminished felt ikigai may be considered in relation to valued roles, relationships, participation, everyday continuity, or opportunities for contribution. Related research links participation, social relationships, and post-disaster environments with mental well-being [3,4,5,20,21,22,23,24,25]; however, none of these potential pathways was measured as a mediator in the present analysis. They remain hypotheses for future work. The two ikigai questions also used different response formats and dichotomisation rules. Their common binary direction permits parallel within-context analyses but not measurement equivalence. The large Okuma binary OR is especially sensitive to coding, as shown by the ordered and boundary-exclusion analyses. Cross-context contrasts in effect magnitude should therefore not be interpreted as differences in an invariant construct.

### 4.4. Limitations

Several limitations materially constrain interpretation. First, all primary analyses were cross-sectional. Psychological distress may reduce perceived ikigai, diminished ikigai may be associated with distress, or both may reflect shared determinants; temporal ordering cannot be established. Second, the primary interaction models were based on 147 Miyakejima and 110 Okuma complete cases. These context-specific community samples match the bounded research settings, but the effective analytic denominators limit precision for interaction estimation, particularly at age-range extremes, and restrict transferability beyond the sampled populations. Interaction tests are data-intensive, and power was particularly limited in Okuma. A nonsignificant interaction therefore indicates insufficient detectable evidence, not evidence that effect modification is absent.

Third, participation may have been selective. Response rates were 49.5% in Miyakejima and 53.6% in Okuma. Miyakejima used postal collection followed months later by in-person distribution and collection, but participant-level mode and period were not retained in the de-identified analytic file; differential participation by age across modes cannot be excluded. Okuma intentionally sampled current returnees. People who remained displaced or relocated may differ systematically in health, resources, and perceived ikigai, so the results do not represent all people originally affected by the Fukushima disaster.

Fourth, missing GHQ-28 data in Okuma and missing ikigai data in Miyakejima reduced analytic denominators. Measured characteristics did not show statistically detected differences between complete and incomplete groups, but these checks do not establish that the missingness mechanism is ignorable. Fifth, the single-item ikigai measures were not psychometrically equivalent across contexts, and dichotomisation discards information. Okuma coding sensitivities demonstrate that estimated effect magnitude varies with representation of the item.

Sixth, residual confounding may be substantial. Health status, social isolation, socioeconomic circumstances, and disaster exposure could influence both perceived ikigai and distress. The one-at-a-time sensitivity models were intended only as perturbation checks, not as a sufficient confounding-control strategy. Seventh, the linear age interaction is a parsimonious approximation; the fixed datasets did not provide sufficient information for highly flexible modelling, and a nonsignificant quadratic test does not establish the linearity assumption. Eighth, secondary and sensitivity analyses were numerous, and *p* values were not adjusted for multiplicity. The possibility that the Miyakejima interaction is a chance finding must therefore remain explicit.

Finally, GHQ high-risk status was common in both samples. Logistic ORs can substantially differ from prevalence ratios when outcomes are common, so the reported ORs should not be interpreted as risk or prevalence ratios. No pooled context × ikigai × age model was fitted; observed differences between Miyakejima and Okuma are descriptive only. The historical Miyakejima comparison is also not longitudinal at the individual level.

### 4.5. Implications and Future Research

The findings do not establish that an ikigai question improves clinical screening, decision-making, or mental-health outcomes. Their immediate implication is analytic: studies interested in age-related heterogeneity should consider testing age continuously rather than relying only on a conventional older-adult threshold. Future research should use larger samples, harmonised and preferably multidimensional meaning measures, planned interaction tests, and longitudinal designs that can establish temporal ordering. Replication across disaster and non-disaster settings is needed before broader ageing or clinical claims are warranted.

## 5. Conclusions

This secondary analysis provides preliminary, context-specific evidence that continuous age may identify heterogeneity in the association between diminished perceived ikigai and psychological distress that a fixed 65-year threshold can miss. The Miyakejima interaction was imprecise; the separately analysed contemporary Okuma and exploratory historical Miyakejima datasets did not provide detectable evidence of a corresponding continuous-age interaction. The result should therefore be regarded as a methodological signal requiring confirmation in larger, preferably longitudinal studies rather than as an established lifespan mechanism.

## Figures and Tables

**Table 1:** Sample Characteristics, Missingness, and Primary Analytic Denominators.

Characteristic	Miyakejima 2020/21	Okuma 2024
Disaster context	Volcanic eruption, island-wide evacuation, return	Earthquake, tsunami, nuclear accident, prolonged evacuation, return
Survey flow	406 distributed; 201 returned (49.5%); 43 ineligible	250 distributed; 134 valid responses (53.6%)
Overall study sample	*N* = 158	*N* = 134
GHQ-28 valid	158/158	111/134; missing *n* = 23
Ikigai valid	147/158; missing *n* = 11	133/134; missing *n* = 1
Primary complete-case model	*n* = 147	*n* = 110
Age, years	M = 68.8; SD = 13.5; range 28–98	M = 63.5; SD = 14.9; range 20–90
Sex data	Valid *n* = 150; missing *n* = 8	Male *n* = 64; female *n* = 70
Descriptive age groups	<65: *n* = 42; ≥65: *n* = 116	<65: *n* = 43; ≥65: *n* = 91
Context-specific diminished ikigai	52/147	65/133; among GHQ-valid complete cases, 53/110
GHQ-28 high risk	81/158 (51.3%)	62/111 (55.9%)

Note. Denominators are shown explicitly because missingness differs by variable and context. Diminished ikigai denotes ikigai absence in Miyakejima and low/absent ikigai in Okuma. The two single-item ikigai measures and their dichotomisation rules are context-specific and are not psychometrically equivalent. GHQ-28 = 28-item General Health Questionnaire; M = mean; SD = standard deviation.

## Data Availability

Participant-level datasets are not publicly available because they contain sensitive disaster-related information from small communities, and public release could create re-identification and ethical risks. De-identified analytic information may be available from the corresponding author on reasonable request, subject to applicable ethical and institutional restrictions.

## Supplementary Materials

The following supporting information can be downloaded at: https://www.mdpi.com/article/10.3390/diseases14100364/s1. Supplementary methods provide variable coding and sampling-frame context. Table S1: Exploratory one-at-a-time perturbation sensitivity checks; Table S2: Missingness checks; Table S3: Directional coding and model treatment of sensitivity covariates; Table S4: Firth bias-reduced continuous-age interaction models in Miyakejima; Table S5: Firth bias-reduced continuous-age models in Okuma; Table S6: Exploratory Firth reanalysis of the 2013/14 Miyakejima survey; Table S7: Contemporary continuous-age model robustness and influence diagnostics; Table S8: Model-based conditional estimates at representative ages in the contemporary surveys; Table S9: Okuma alternative ikigai-coding sensitivity; Table S10: Secondary <65/≥65 interaction models; Table S11: Observed age distributions by context-specific ikigai status in the primary complete-case samples.
