# Supplementary material for: Testing Continuous Age as an Effect Modifier of the Association Between Diminished Ikigai and Psychological Distress in Two Long-Term Disaster Contexts in Japan
Source: Diseases. 2026 Oct 2;14(10):364. doi: 10.3390/diseases14100364 (PMC13703247; doi:10.3390/diseases14100364)
Supplement: Supplementary file 1 [file diseases-14-00364-s001.zip › diseases-4539122-supplementary.pdf]

Supplementary Material

Testing Continuous Age as an Effect Modifier of the Association Between Diminished Ikigai and Psychological Distress in Two Long-Term Disaster Contexts in Japan

Supplementary methods: variable coding

Focal variables

General Health Questionnaire-28 (GHQ-28) items were scored using the standard 0-0-1-1 method. Total scores ranged from 0 to 28, with scores of 6 or higher classified as high risk. Analyses used complete GHQ-28 responses without imputation or prorating. In Miyakejima, the item "Do you feel a sense of ikigai in your current daily life?" used four frequency categories (often, sometimes, seldom, and not at all). Often/sometimes were classified as present and seldom/not at all as absent. In Okuma, the six ordered categories were strongly feel, feel, somewhat feel, do not feel much, do not feel, and do not feel at all. The first three were classified as present and the latter three as low or absent in the primary binary analysis.

Age was recorded in years. In both contemporary datasets, the primary model used continuous age centred at 65 years and scaled per 10 years. The continuous interaction therefore represents the multiplicative change in the context-specific diminished-ikigai odds ratio (OR) for each 10-year increase in age. Secondary <65/≥65 variables were derived directly from the age values (Miyakejima: 42 aged <65 years and 116 aged ≥65 years; Okuma: 43 aged <65 years and 91 aged ≥65 years). The archived 2013/14 Miyakejima reanalysis retained the <65/≥65 grouping for comparability with predecessor evidence. A Firth continuous-age model was added as a post hoc historical sensitivity, not as a prespecified replication.

Sampling-frame context

The Okuma source survey was restricted to adults aged ≥20 years who had lived in Okuma on 11 March 2011 and had returned to reside in the town by 2024. Questionnaires were distributed door to door to 250 adults and 134 valid responses were obtained. Former residents who remained displaced or relocated were outside the sampling frame. Municipal statistics recorded 269 residents in the administrative returnee category on 31 May and 282 on 30 September 2024. These counts contextualise the scale of the contemporaneous returnee population. They are not used as a sampling denominator because administrative and study eligibility definitions differ.

Miyakejima collection-mode information. Postal fieldwork was conducted from 28 October to 30 November 2020, followed by in-person distribution and collection on 16–18 March and 3–6 April 2021 because postal return was limited. Participant-level collection mode and period were not retained in the de-identified analytic file; therefore, a valid comparison of age distributions by collection mode could not be performed. Differential participation by age across modes remains a limitation.

Sensitivity covariates

Exploratory perturbation analyses added one source-survey covariate at a time to the context-specific interaction model. These models were not treated as a sufficient confounding-control strategy. The covariates were retained because prior disaster and ageing research identifies health, socioeconomic circumstances, social relationships, participation, and disaster-related concerns as plausible common causes or contextual correlates of perceived ikigai and psychological distress. Miyakejima covariates were sex, income change, living conditions, health anxiety, natural-disaster anxiety, stress, weak neighbourhood ties, no social activity, and life dissatisfaction. Okuma covariates were sex, income change, living conditions, health anxiety, current health, community exchange, consultation partner, life satisfaction, future anxiety, radiation anxiety, and return anxiety. Covariates retained their original binary or ordered coding, preserving context-specific metrics. Supplementary Table S3 reports the substantive direction represented by higher-risk or less favourable values and the model-treatment principle.

Miyakejima continuous-age robustness analyses

The primary Miyakejima complete-case model (n = 147) included ikigai absence, continuous age, and their interaction. Age was centred at 65 years and scaled per 10 years. The interaction model was compared with an additive model using a likelihood-ratio test (LRT). Firth bias-reduced logistic regression assessed finite-sample bias. A second Firth model added sex. One-at-a-time perturbation models assessed directional stability of the age interaction; they were not interpreted as formal confounding control. A quadratic extension added age squared and its interaction with ikigai. Cook's distance and leave-one-out refitting assessed influence. Conditional ikigai ORs and predicted probabilities were calculated at ages 55, 65, 75, and 85 years.

Okuma continuous-age robustness analyses

The primary Okuma complete-case model (n = 110) included low or absent ikigai, continuous age, and their interaction. Age was centred at 65 years and scaled per 10 years. The interaction model was compared with an additive model using an LRT. Firth interaction models assessed finite-sample bias, with a second model adding sex. Additive Firth models assessed the overall ikigai association. One-at-a-time covariate models retained source binary or ordered coding. A quadratic extension added age squared and its interaction with ikigai. Cook's distance and leave-one-out refitting assessed influence. Conditional ikigai ORs and predicted probabilities were calculated at ages 55, 65, 75, and 85 years. The core robustness analyses used R version 4.5.1 (R Foundation for Statistical Computing, Vienna, Austria) and the logistf package version 1.26.1; profile penalised-likelihood confidence intervals and penalised-likelihood-ratio p values are reported for Firth models.

Okuma alternative ikigai-coding sensitivity. Two sensitivities examined whether results depended on the primary 1-3 versus 4-6 boundary. First, the full six-category response was retained as a one-step ordered predictor (higher = less felt ikigai) in additive and continuous-age interaction models using all 110 complete cases. Second, a boundary-exclusion analysis omitted category 3 (‘somewhat feel’) and compared categories 1-2 (‘strongly feel’/‘feel’) with categories 4-6 (the three negative responses), yielding n = 84. Both used age centred at 65 years and scaled per 10 years. The boundary-exclusion analysis avoids forcing the intermediate affirmative response into either comparison group.

Exploratory historical Miyakejima reanalysis

The archived 2013/14 Miyakejima survey provided an exploratory historical check of the 2020/21 age pattern. The archive contained 204 respondents with age in years, GHQ-28 high-risk status, sex, and a four-category felt-ikigai response harmonised in the same binary direction as in 2020/21. Age was categorised as <65 versus ≥65 years to match the predecessor framing. As a post hoc sensitivity, continuous age was centred at 65 years, scaled per 10 years, and entered in Firth interaction models with and without sex adjustment. All 17 respondents aged ≥65 years without ikigai were GHQ high risk, producing complete separation under conventional maximum-likelihood logistic regression. Firth bias-reduced regression was therefore used for all historical regression models. The 2013/14 and 2020/21 surveys were unlinked repeated cross-sectional samples. The comparison does not estimate within-person change.

Supplementary Table S1. Exploratory one-at-a-time perturbation sensitivity checks

Panel A. Miyakejima continuous-age interaction. Each model added one covariate to GHQ high-risk status ~ ikigai absence × age (per 10 years, centred at 65). The table reports the conditional ikigai OR at age 65 and the interaction OR per 10-year increase in age. LRT p values compare the interaction model with the corresponding additive covariate model.

Covariate added	*N*	Ikigai OR at 65 [95% CI]	p	Interaction OR /10y [95% CI]	Wald p	LRT p
Base model (none)	147	1.23 [0.58, 2.57]	0.590	2.00 [1.03, 3.91]	0.042	0.029
Sex	141	1.07 [0.47, 2.44]	0.867	2.03 [1.04, 3.97]	0.039	0.027
Income change	134	1.43 [0.65, 3.15]	0.374	2.59 [1.20, 5.62]	0.016	0.007
Living conditions	135	1.54 [0.70, 3.40]	0.282	1.97 [1.00, 3.87]	0.050	0.036
Health anxiety	145	1.28 [0.60, 2.70]	0.522	1.96 [1.01, 3.83]	0.048	0.035
Natural-disaster anxiety	145	1.24 [0.58, 2.63]	0.581	1.96 [1.00, 3.84]	0.049	0.036
Stress	146	1.28 [0.57, 2.90]	0.547	2.04 [1.03, 4.02]	0.040	0.027
Weak neighbourhood ties	145	0.96 [0.43, 2.14]	0.921	2.13 [1.08, 4.20]	0.029	0.019
No social activity	147	1.15 [0.54, 2.45]	0.726	1.92 [0.98, 3.75]	0.058	0.044
Life dissatisfaction	147	1.06 [0.45, 2.49]	0.892	2.02 [1.04, 3.93]	0.038	0.026

Supplementary Table S1 (continued)

Panel B. Okuma continuous-age interaction. Each model added one available covariate to GHQ high-risk status ~ low or absent ikigai × age (per 10 years, centred at 65). The table reports the conditional low/absent-ikigai OR at age 65 and the interaction OR per 10-year increase in age. LRT p values compare the interaction model with the corresponding additive covariate model.

Covariate added	*N*	Ikigai OR at 65 [95% CI]	p	Interaction OR /10y [95% CI]	Wald p	LRT p
Base model (none)	110	12.72 [4.87, 33.25]	< 0.001	0.88 [0.44, 1.76]	0.711	0.708
Sex	110	12.62 [4.81, 33.10]	< 0.001	0.87 [0.43, 1.76]	0.705	0.702
Income change	99	11.54 [3.98, 33.49]	< 0.001	0.69 [0.27, 1.74]	0.430	0.429
Living conditions	106	11.88 [4.29, 32.91]	< 0.001	1.01 [0.46, 2.22]	0.977	0.977
Health anxiety	105	10.89 [4.12, 28.82]	< 0.001	0.82 [0.41, 1.63]	0.569	0.566
Current health	110	11.05 [4.16, 29.32]	< 0.001	0.81 [0.40, 1.68]	0.577	0.571
Community exchange	109	10.84 [3.76, 31.23]	< 0.001	0.85 [0.42, 1.72]	0.657	0.654
Consultation partner	110	7.71 [2.78, 21.40]	< 0.001	0.68 [0.32, 1.41]	0.298	0.289
Life satisfaction	110	5.89 [1.87, 18.54]	0.002	0.84 [0.41, 1.75]	0.650	0.647
Future anxiety	101	11.64 [4.24, 31.92]	< 0.001	0.84 [0.41, 1.73]	0.642	0.638
Radiation anxiety	104	11.01 [4.18, 29.03]	< 0.001	0.88 [0.44, 1.74]	0.707	0.705
Return anxiety	101	10.49 [3.91, 28.13]	< 0.001	0.80 [0.39, 1.64]	0.539	0.533

Note. In both panels, interaction ORs represent the multiplicative change in the context-specific diminished-ikigai OR per 10-year increase in age. These one-at-a-time models are exploratory perturbation checks, not a confounding-control strategy. P values are unadjusted; the analyses should not be interpreted as independent replications. Life satisfaction in Okuma is conceptually close to ikigai and may represent overadjustment. OR = odds ratio; CI = confidence interval; LRT = likelihood-ratio test; GHQ-28 = 28-item General Health Questionnaire.

Supplementary Table S2. Missingness checks

Panel A. Okuma GHQ missingness check

Variable	Valid GHQ data	Missing GHQ data	p
Male	55/111 (49.5%)	9/23 (39.1%)	0.492
Female	56/111 (50.5%)	14/23 (60.9%)	
<65 years	39/111 (35.1%)	4/23 (17.4%)	0.140
≥65 years	72/111 (64.9%)	19/23 (82.6%)	
Age, years	M = 62.9; SD = 15.1; median = 66; range 20-90	M = 66.3; SD = 14.0; median = 68; range 28-82	Wilcoxon p = 0.202
Ikigai present	57/110 (51.8%)	11/23 (47.8%)	0.820
Low/absent ikigai	53/110 (48.2%)	12/23 (52.2%)	

Panel B. Miyakejima ikigai missingness check

Variable	Valid ikigai data	Missing ikigai data	p
GHQ lower risk	70/147 (47.6%)	7/11 (63.6%)	0.360
GHQ high risk	77/147 (52.4%)	4/11 (36.4%)	
Sex code 1	67/141 (47.5%)	5/9 (55.6%)	0.738
Sex code 2	74/141 (52.5%)	4/9 (44.4%)	
Age, years	M = 68.6; SD = 13.3; median = 72; range 28-94	M = 71.2; SD = 16.4; median = 75; range 38-98	Wilcoxon p = 0.479

Note. Okuma GHQ missingness was assessed using Fisher exact tests for categorical variables and a Wilcoxon rank-sum test for continuous age. Miyakejima ikigai missingness was assessed using the same approach. No statistically detected differences were observed on the measured characteristics, but nonsignificant comparisons do not establish that the missingness mechanism is ignorable or missing at random. GHQ-28 = 28-item General Health Questionnaire; M = mean; SD = standard deviation.

Supplementary Table S3. Directional coding and model treatment of sensitivity covariates

Context	Covariate	Less favourable direction	Model treatment
Miyakejima	Sex	Survey-specific binary sex variable	Binary adjustment variable; coefficient not interpreted
Miyakejima	Income change	Greater income decrease or worsening	Source coding retained; higher = less favourable
Miyakejima	Living conditions	Less favourable or worsened living conditions	Source coding retained; higher = less favourable
Miyakejima	Health anxiety	Greater health-related anxiety	Source coding retained; higher = less favourable
Miyakejima	Natural-disaster anxiety	Greater anxiety about natural disaster	Source coding retained; higher = less favourable
Miyakejima	Stress	Stress present or greater stress	Binary: less favourable vs favourable
Miyakejima	Weak neighbourhood ties	Weaker or absent neighbourhood interaction	Source coding retained; higher = less favourable
Miyakejima	No social activity	No participation in social activity	Binary: less favourable vs favourable
Miyakejima	Life dissatisfaction	Lower life satisfaction / greater dissatisfaction	Source coding retained; higher = less favourable
Okuma	Sex	Survey-specific binary sex variable	Binary; source reference retained
Okuma	Income change	Greater income decrease or worsening	Source coding retained; higher = less favourable
Okuma	Living conditions	Less favourable or worsened living conditions	Source coding retained; higher = less favourable
Okuma	Health anxiety	Greater own/family health anxiety	Source coding retained; higher = less favourable
Okuma	Current health	Poorer self-rated current health	Source coding retained; higher = less favourable
Okuma	Community exchange	Weaker community/neighbourhood exchange	Source coding retained; higher = less favourable
Okuma	Consultation partner	No person available for consultation	Binary: less favourable vs favourable
Okuma	Life satisfaction	Lower life satisfaction	Source coding retained; higher = less favourable
Okuma	Future anxiety	Greater anxiety about the future of Okuma	Source coding retained; higher = less favourable
Okuma	Radiation anxiety	Greater radiation-related health anxiety	Source coding retained; higher = less favourable
Okuma	Return anxiety	Greater return/resettlement anxiety	Source coding retained; higher = less favourable

Note. Exact numerical labels were retained from each source survey and were not harmonised across contexts. Survey-specific sex coding was used only as an adjustment variable; its coefficient was not interpreted.

Supplementary Table S4. Firth bias-reduced continuous-age interaction models in Miyakejima

Model	Predictor	OR	95% profile penalised-likelihood CI	PLR p
Base Firth model	Ikigai absence at age 65	1.24	[0.60, 2.57]	0.555
Base Firth model	Age, per 10 years, among ikigai-present	0.88	[0.66, 1.15]	0.354
Base Firth model	Ikigai absence × age, per 10 years	1.88	[1.04, 3.80]	0.037
Age- and sex-adjusted Firth model	Ikigai absence at age 65	1.09	[0.49, 2.43]	0.828
Age- and sex-adjusted Firth model	Age, per 10 years, among ikigai-present	0.87	[0.66, 1.15]	0.334
Age- and sex-adjusted Firth model	Sex code	0.87	[0.42, 1.82]	0.714
Age- and sex-adjusted Firth model	Ikigai absence × age, per 10 years	1.90	[1.05, 3.83]	0.035

Note. The base model used *n* = 147 complete cases; the sex-adjusted model used *n* = 141. Age was centred at 65 years and scaled per 10 years. Confidence intervals and p values are profile penalised-likelihood quantities. Firth estimation addresses separation and finite-sample estimation bias but does not resolve limited power, selection bias, residual confounding, or measurement error. OR = odds ratio; CI = confidence interval; PLR = penalised-likelihood-ratio test.

Supplementary Table S5. Firth bias-reduced continuous-age models in Okuma

Model	Predictor	OR	95% profile penalised-likelihood CI	PLR p
Additive Firth model	Low or absent ikigai	11.8	[4.83, 31.5]	< 0.001
Additive Firth model	Age, per 10 years	1.26	[0.93, 1.76]	0.136
Additive + sex Firth model	Low or absent ikigai	11.4	[4.68, 30.3]	< 0.001
Additive + sex Firth model	Age, per 10 years	1.27	[0.93, 1.78]	0.138
Additive + sex Firth model	Female	0.93	[0.37, 2.36]	0.877
Interaction Firth model	Low or absent ikigai at age 65	11.3	[4.63, 29.9]	< 0.001
Interaction Firth model	Age, per 10 years, among ikigai-present	1.30	[0.90, 2.02]	0.168
Interaction Firth model	Low or absent ikigai × age, per 10 years	0.92	[0.46, 1.74]	0.799
Interaction + sex Firth model	Low or absent ikigai at age 65	10.9	[4.49, 28.7]	< 0.001
Interaction + sex Firth model	Age, per 10 years, among ikigai-present	1.30	[0.90, 2.04]	0.168
Interaction + sex Firth model	Female	0.93	[0.37, 2.34]	0.868
Interaction + sex Firth model	Low or absent ikigai × age, per 10 years	0.92	[0.45, 1.73]	0.792

Note. All Okuma models used n = 110. Age was centred at 65 years and scaled per 10 years. Confidence intervals and p values are profile penalised-likelihood quantities. Additive models quantify the overall low/absent-ikigai association when no age interaction is included; interaction-model ikigai effects are conditional at age 65. Firth estimation does not overcome limited power, selection bias, residual confounding, or measurement error. OR = odds ratio; CI = confidence interval; PLR = penalised-likelihood-ratio test.

Supplementary Table S6. Exploratory Firth reanalysis of the 2013/14 Miyakejima survey

Age group	Ikigai	*N*	GHQ high risk	GHQ lower risk	High-risk rate
<65 years	Present	73	16	57	21.9%
<65 years	Absent	14	13	1	92.9%
≥65 years	Present	100	57	43	57.0%
≥65 years	Absent	17	17	0	100.0%

Model	Predictor	OR	95% profile penalised-likelihood CI	PLR p
Age-stratified: <65 years	Ikigai absence	31.36	[6.86, 302.26]	< 0.001
Age-stratified: ≥65 years	Ikigai absence	26.48	[3.43, 3407.95]	< 0.001
Interaction model	Ikigai absence × age ≥65 years	0.84	[0.04, 131.04]	0.922
Sex-adjusted interaction model	Ikigai absence among <65 years	28.64	[6.07, 280.78]	< 0.001
Sex-adjusted interaction model	Age ≥65 years among ikigai-present	5.26	[2.65, 10.96]	< 0.001
Sex-adjusted interaction model	Female	2.93	[1.52, 5.79]	0.001
Sex-adjusted interaction model	Ikigai absence × age ≥65 years	0.90	[0.04, 140.70]	0.950
Continuous-age Firth model	Ikigai absence at age 65	37.77	[7.85, 937.31]	< 0.001
Continuous-age Firth model	Age, per 10 years, among ikigai-present	1.82	[1.38, 2.45]	< 0.001
Continuous-age Firth model	Ikigai absence × age, per 10 years	0.85	[0.36, 2.23]	0.703
Continuous-age + sex Firth model	Ikigai absence at age 65	37.93	[7.56, 1077.79]	< 0.001
Continuous-age + sex Firth model	Age, per 10 years, among ikigai-present	1.95	[1.47, 2.68]	< 0.001
Continuous-age + sex Firth model	Female	3.15	[1.63, 6.27]	< 0.001
Continuous-age + sex Firth model	Ikigai absence × age, per 10 years	0.88	[0.36, 2.41]	0.771

Note. $N = 204$. Threshold models used age <65 years and ikigai present as reference categories. Continuous-age Firth models centred age at 65 years and scaled it per 10 years. In the continuous-age models, interaction ORs represent the multiplicative change in the ikigai-absence OR per 10-year increase in age. Complete separation occurred in the ≥65-years/ikigai-absent stratum because all 17 respondents were GHQ high risk. The continuous-age models were post hoc historical sensitivities. Because the 2013/14 and 2020/21 samples were unlinked, these analyses do not estimate within-person change or provide a confirmatory temporal replication. GHQ-28 = 28-item General Health Questionnaire; OR = odds ratio; CI = confidence interval; PLR = penalised-likelihood-ratio test.

Supplementary Table S7. Contemporary continuous-age model robustness and influence diagnostics

Context	Check	Result	Interpretation
Miyakejima	Interaction vs additive LRT	Δdeviance = 4.74; df = 1; p = 0.029	Interaction improved fit
Miyakejima	Quadratic extension LRT	Δdeviance = 1.05; df = 2; p = 0.591	No evidence that quadratic age terms improved fit
Miyakejima	Cook's distance	3/147 cases > 4/*n*; maximum = 0.156	No Cook's distance approached 1
Miyakejima	Leave-one-out refitting	Interaction OR range = 1.87-2.60; p range = 0.015-0.084; 129/147 p < 0.05	Direction preserved in all 147 models
Miyakejima	Largest Cook's-distance case omitted	Interaction OR = 2.60; 95% CI [1.20, 5.60]; p = 0.015	Interaction did not depend on the most influential case
Okuma	Interaction vs additive LRT	Δdeviance = 0.14; df = 1; p = 0.708	Interaction did not improve fit
Okuma	Quadratic extension LRT	Δdeviance = 0.12; df = 2; p = 0.941	No evidence that quadratic age terms improved fit
Okuma	Cook's distance	6/110 cases > 4/*n*; maximum = 0.198	No Cook's distance approached 1
Okuma	Leave-one-out refitting	Interaction OR range = 0.662-1.07; p range = 0.323-0.979; 0/110 p < 0.05	No leave-one-out refit supported an interaction
Okuma	Largest Cook's-distance case omitted	Interaction OR = 0.662; 95% CI [0.292, 1.50]; p = 0.323	Conclusion remained unchanged

Note. Influence diagnostics were used as robustness checks rather than additional hypothesis tests. Leave-one-out analyses removed each complete case once and refitted the same maximum-likelihood interaction model within each context. LRT = likelihood-ratio test; df = degrees of freedom; OR = odds ratio; CI = confidence interval.

Supplementary Table S8. Model-based conditional estimates at representative ages in the contemporary surveys

Context	Age	Diminished -ikigai OR	95% CI	p	Predicted GHQ risk: present	Predicted GHQ risk: diminished
Miyakejima	55	0.61	[0.19, 1.92]	0.401	52.9% [39.2%, 66.2%]	40.8% [20.2%, 65.2%]
Miyakejima	65	1.23	[0.58, 2.57]	0.590	49.6% [39.4%, 59.9%]	54.7% [39.5%, 69.0%]
Miyakejima	75	2.45	[1.07, 5.61]	0.033	46.2% [35.6%, 57.3%]	67.8% [51.2%, 80.9%]
Miyakejima	85	4.91	[1.33, 18.17]	0.017	42.9% [28.9%, 58.2%]	78.7% [53.8%, 92.1%]
Okuma	55	14.5	[4.59, 45.9]	< 0.001	24.6% [14.2%, 39.0%]	82.5% [65.0%, 92.3%]
Okuma	65	12.7	[4.87, 33.2]	< 0.001	30.4% [19.4%, 44.2%]	84.8% [72.3%, 92.2%]
Okuma	75	11.1	[3.27, 37.9]	< 0.001	37.0% [21.3%, 56.0%]	86.8% [71.7%, 94.4%]
Okuma	85	9.76	[1.70, 56.0]	0.011	44.1% [21.0%, 70.1%]	88.5% [66.3%, 96.8%]

Note. Estimates are descriptive model-based illustrations from the context-specific continuous-age interaction models. Ages 55, 65, 75, and 85 are not thresholds and the age-specific p values are not independent hypothesis tests. Precision decreases at age-range extremes. Diminished ikigai means absent in Miyakejima and low/absent in Okuma. Because the Okuma interaction was unsupported, its interaction-model representative-age estimates should not be read as evidence of age modification; the main-text Figure 2 uses the additive model instead. GHQ-28 = 28-item General Health Questionnaire; OR = odds ratio; CI = confidence interval.

Supplementary Table S9. Okuma alternative ikigai-coding sensitivity

Specification	Focal estimate	OR / test	95% CI	p	Interaction LRT p
Primary binary additive	Low/absent (4-6) vs present (1-3)	12.94	[4.96, 33.76]	< 0.001	—
Primary binary interaction	Low/absent × age, per 10 years	0.88	[0.44, 1.76]	0.711	0.708
Six-category ordered additive	Per one-category shift toward less felt ikigai	3.28	[2.01, 5.33]	< 0.001	—
Six-category ordered interaction	Ikigai score × age, per 10 years	0.94	[0.67, 1.31]	0.717	0.715
Boundary-exclusion additive	Negative (4-6) vs clear affirmative (1-2)	20.57	[6.20, 68.27]	< 0.001	—
Boundary-exclusion interaction	Negative vs affirmative × age, per 10 years	1.02	[0.45, 2.30]	0.960	0.961

Note. Primary binary: categories 1-3 versus 4-6 (n = 110). Ordered sensitivity: all six categories, higher = less felt ikigai (n = 110). Boundary-exclusion: category 3 (‘somewhat feel’) omitted; categories 1-2 versus 4-6 (n = 84). Interaction ORs are per 10-year age increase. Both sensitivities supported no detectable age modification. OR = odds ratio; CI = confidence interval; LRT = likelihood-ratio test.

Supplementary Table S10. Secondary <65/≥65 interaction models

Context	Predictor	OR	95% CI	Wald p	Interaction LRT p
Miyakejima	Ikigai absence among adults aged <65	0.92	[0.25, 3.42]	0.905	
Miyakejima	Age ≥65 among ikigai-present	0.82	[0.33, 2.05]	0.677	
Miyakejima	Ikigai absence × age ≥65	2.08	[0.45, 9.70]	0.350	0.350
Okuma	Low/absent ikigai among adults aged <65	11.61	[2.41, 55.94]	0.002	
Okuma	Age ≥65 among ikigai-present	1.44	[0.44, 4.70]	0.546	
Okuma	Low/absent ikigai × age ≥65	1.29	[0.18, 9.31]	0.802	0.802

Note. These threshold models are secondary. Small subgroup cells and wide confidence intervals limit precision; the interaction tests do not support an abrupt change at age 65 in either contemporary context. OR = odds ratio; CI = confidence interval; LRT = likelihood-ratio test.

Supplementary Table S11. Observed age distributions by context-specific ikigai status in the primary complete-case samples

Context	Ikigai status	*n*	Mean (SD)	Median	IQR	Range	*n* ≥75	*n* ≥85
Miyakejima	Ikigai present	95	68.5 (14.6)	72	64–76	28–92	31	9
Miyakejima	Ikigai absent	52	68.9 (10.8)	71	64–74.25	29–94	13	3
Okuma	Ikigai present	57	59.4 (16.4)	66	50–72	20–81	9	0
Okuma	Low/absent ikigai	53	66.6 (12.7)	68	63–72	21–90	11	4

Note. These distributions are from the primary complete-case samples. Sparse upper-age observations, especially in Okuma, increase uncertainty. Main-text rug marks show observed age density using legend-matched colours; lower row = ikigai present and upper row = diminished/low-absent ikigai. SD = standard deviation; IQR = interquartile range.
